# Communication strategies in the prevention of type 2 and gestational diabetes in vulnerable groups: protocol for a scoping review

**DOI:** 10.1186/s13643-019-1021-9

**Published:** 2019-04-18

**Authors:** Jessica Breuing, Christine Graf, Annika Lena Neuhaus, Simone Heß, Lena Lütkemeier, Fabiola Haas, Mark Spiller, Dawid Pieper

**Affiliations:** 1 Institute for Research in Operative Medicine (IFOM), Faculty of Health, Department of Medicine, Witten/Herdecke University, Ostmerheimer Str. 200, Building 38, 51109 Cologne, Germany; 2 Institute of Movement and Neurosciences, German Sport University Cologne, Am Sportpark Müngersdorf 6, 50933 Cologne, Germany

**Keywords:** Diabetes mellitus type 2, Prevention, Vulnerable groups, Communication strategies

## Abstract

**Background:**

The global prevalence of diabetes mellitus is nearly 9%, with an upward trend in type 2 and gestational diabetes mellitus (T2DM/GDM). Evidence shows that vulnerable groups are affected disproportionally. Therefore, there is an increasing need to implement policies to prevent risk factors for T2DM/GDM and to promote a healthy lifestyle. However, up to now, no gold standard in terms of communication strategies and/or public awareness campaigns is known.

**Methods/design:**

We will conduct a systematic scoping review to evaluate communication strategies in the prevention of T2DM/GDM in vulnerable groups. Two reviewers will independently screen the results of the electronic literature search in PubMed, EMBASE, PsycINFO, PSYNDEX, Social Science Citation Index, and CINAHL. Extracted data will be charted, categorized, and summarized.

**Discussion:**

The results will be used to inform the National education and communication strategy on diabetes mellitus in Germany. In particular, the results will be discussed in focus groups of experts to develop recommendations for communication strategies.

**Systematic review registration:**

PROSPERO does not register scoping reviews.

## Background

The global prevalence of diabetes mellitus is nearly 9% [1], with 90% of patients having type 2 diabetes mellitus (T2DM). Additionally, the prevalence of gestational diabetes mellitus (GDM) is increasing with about 16% of all live births being affected by hyperglycemia [2]. Because of its health consequences, the global health-related costs are expected to nearly double from US $1.3 trillion in 2015 to $2.5 trillion by 2030, taking past trends into account [3]. This equals an increase in costs as a share of global gross domestic product (GDP) from 1.8% in 2015 to a maximum of 2.2% in 2030 [3].

Many cases of T2DM and GDM could be prevented with lifestyle changes, including maintaining a healthy body weight, consuming a healthy diet and staying physically active [4]. Therefore, there is an increasing need to implement effective preventive policies and to promote a healthy lifestyle.

Ethnicity and/or lower socio-economic status are important considerations in individuals affected by diabetes. For example, people in the lowest socio-economic groups are 2.5 times as likely, and black and minority ethnic groups up to six times as likely, to develop diabetes compared with the general population [5]. This could partly be attributed to lifestyle factors, e.g., obesity, which more severely affect deprived communities and those living in vulnerable circumstances [6]. Yet they are even harder to reach in terms of preventive measures [6].

Numerous studies demonstrated that T2DM can be prevented or delayed by intensive lifestyle changes in individuals with pre-diabetes [7]. However, little is known in terms of effective communication or awareness strategies in primary prevention of T2DM or GDM, in particular regarding accessibility to those who are hardest to reach and most at risk. Identifying barriers and facilitators is necessary to increase the number of participants in a preventive intervention addressing vulnerable groups. But just as important as this, we must determine communication strategies as well to get access to participants especially in vulnerable groups. Therefore, we aimed to identify, e.g., translations or modifications of existing programs or new communication strategies for vulnerable groups. Our target audiences are primary care providers (e.g., general practitioners, nutritionists, and midwifes) as well as diabetologists and public health experts active in diabetes prevention. The aim of this study is to systematically review the literature in order to identify and describe communication strategies in the prevention of T2DM/GDM in vulnerable groups.

## Methods

This project was commissioned by Federal Centre for Health Education in Germany as part of the “National education and communication strategy on diabetes mellitus”. This is one of two scoping reviews, both of which will use the same search strategy and are similar in their methodology. In this scoping review, we will focus on communication strategies, and in the other, we will review barriers and facilitators for participating in preventative interventions aimed at vulnerable participants with, or at risk of, T2DM/GDM.

### Protocol

This protocol was established according to PRISMA-P guidelines [8]. The scoping review will be conducted following the Arksey and O’Malleys framework and The Joanna Briggs Institute Reviewers’ Manual 2015 [9, 10].

### Eligibility criteria

#### Inclusion criteria

- Vulnerable patients with, or at risk of T2DM or GDM
- Studies present for implementing a communication strategies for the prevention of T2DM/GDM
- WHO mortality stratum A countries
- Publication date ≥ 2008

#### Exclusion criteria

- Native people, children, or people with mental disorders (e.g., schizophrenia, bipolar disorders)
- No full texts available

Eligibility criteria are shown in the PPC (Population, Concept, Context) mnemonic in Table 1. We will include studies presenting communication strategies for the prevention of T2DM/GDM in vulnerable groups. We will exclude people with mental disorders, e.g., schizophrenia or bipolar disorders. We assume that for this type of mental disorder, other communication strategies are needed compared to the included vulnerable groups. In case we identify articles regarding homeless people, we will check if any mental disorders are described or mentioned in the inclusion criteria. If so, we will exclude this article. We will not exclude people with drug addiction per se because we suspect high rates of drug addiction within the vulnerable group of homeless people. Publications will be restricted to studies published from January 2008 onwards. Communication strategies are affected by external factors such as accessibility of care and information. We assume that there has been a change in accessibility due to the volume of digital and virtual goods, services, and processes in healthcare over the past 10 years. As a result, communication strategies might have changed, so that there would be a lack of comparability if we chose a longer period. No language restrictions will be made. All full texts published in languages other than English or German will be translated by an external agency. Furthermore, we will only include studies with a low mortality stratum (A) according to the World Health Organization (WHO) [11]. WHO stratum A indicates countries with very low child mortality and low adult mortality. By doing so, we will ensure that our findings will be applicable to western industrialized countries. We define vulnerable groups using the definition of Lewis et al. [12] (Table 1: Population, Concept, Context (PCC)). Unlike Lewis’ definition, we exclude native people, children, and people with mental disorders. Since Germany does not have a native population similar to the USA, Canada, or South and Middle American countries, we decided to exclude studies focusing on native individuals. Children and individuals with mental disorders (e.g., schizophrenia or bipolar disorders) seem to need other communication strategies because you have to address their caregivers; therefore, we excluded these individuals.

**Table 1:** PCC (Population, Concept, Context)

P	Diabetes mellitus type 2 or gestation diabetes	Type 2 diabetes mellitus Gestational diabetes mellitus People at risk of developing diabetes mellitus or gestational diabetes mellitus
	Vulnerable patients/groups	Elderly, older people, seniors > 65 years Disabled people People in need of care, residents of a nursing home Unemployed people Refugees/migrants as well as ethnic groups (e.g., African Americans or Hispanics) Homeless people Drug/substance abusers (excluding nicotine abuse/smoking) Low socio-economic status
C	Prevention	Primary/secondary prevention
	Communication strategies	Communication strategies/ access routes, e.g., digital/social media, TV/radio, print media, group sessions, health campaign
C	> 2008; WHO stratum A	
Other	All types of studies; all languages; available in full text version	

The PPC (Population, Concept, Context) mnemonic illustrates the eligibility criteria for the scoping review. Additionally to the classic PPC mnemonic, there are other criteria regarding study types, languages, and the availability of the full text version

### Information sources

The following electronical databases will be searched: PubMed, EMBASE, PsycINFO, PSYNDEX, Social Science Citation Index, and CINAHL. Grey literature will be searched in greylit.org and through the homepages of the WHO and international, health care, or public health departments (e.g., Department of Health & Social Care, UK; Agency for Healthcare Research and Quality (AHRQ); US Preventive Services Task Force). We will search manually for additional studies by cross-checking the reference lists of all included studies.

### Search

The search strategy will be developed by the research team in collaboration with an experienced librarian and checked by a referee according to the Peer Review of Electronic Search Strategies (PRESS) guideline [13]. As an example, we present the search strategy which will be used in PubMed (Table 2).

**Table 2:** Search strategy for PubMed

	“Diabetes, Gestational”[Mesh] OR “Diabetes Mellitus, Type 2”[Mesh] OR gestational diabetes[tiab] OR diabetes mellitus, gestational[tiab] OR pregnancy-induced diabetes[tiab] OR type 2 diabetes[tiab] OR “diabetes mellitus type II”[tiab] OR type 2 diabetes mellitus[tiab] OR “diabetes type 2”[tiab]
AND	(“Ethnic Groups”[Mesh] OR “Minority Groups”[Mesh] OR “Poverty Areas”[Mesh] OR “Vulnerable Populations”[Mesh] OR “Health Status Disparities”[Mesh] OR “Cultural Diversity”[Mesh] OR “Socioeconomic Factors”[Mesh] OR “Aged”[Mesh] OR “Substance-Related Disorders”[Mesh] OR “Malnutrition”[Mesh] OR “Disabled Persons”[Mesh] OR “Educational Status”[Mesh] OR “Emigrants and Immigrants”[Mesh] OR “Homeless Persons”[Mesh] OR “Minors”[Mesh] OR “Transients and Migrants”[Mesh] OR “Refugees”[Mesh] OR “Unemployment”[Mesh] OR “Mental Disorders”[Mesh] OR ethnic group*[tiab] OR ethnic population*[tiab] OR minority[tiab] OR minorities[tiab] OR ethnic minorit*[tiab] OR poverty[tiab] OR destitution[tiab] OR poor housing[tiab] OR addiction[tiab] OR drug abuse[tiab] OR malnutrition[tiab] OR malnourished[tiab] OR vulnerable population*[tiab] OR vulnerable group*[tiab] OR socioeconomic factor*[tiab] OR socioeconomic aspect*[tiab] OR deprived[tiab] OR health status[tiab] OR aged[tiab] OR elderly[tiab] OR elders[tiab] OR minors[tiab] OR disabled[tiab] OR disability[tiab] OR level of education[tiab] OR education level[tiab] OR mental disorder[tiab] OR need for care[tiab] OR need of care[tiab] OR care dependency[tiab] OR unemployment[tiab] OR ethnic disparity[tiab] OR ethnic disparities[tiab] OR migrant[tiab] OR migrants[tiab] OR immigrant[tiab] OR immigrants[tiab] OR asylum[tiab] OR refugees[tiab] OR cultural diversity[tiab] OR multicultural aspect*[tiab] OR multicultural factor*[tiab] OR religion[tiab] OR homeless*[tiab])
AND	(“Primary Prevention”[Mesh] OR “Secondary Prevention”[Mesh] OR “Tertiary Prevention”[Mesh] OR “Preventive Health Services”[Mesh] OR “Mass Screening”[Mesh] OR “Health Promotion”[Mesh] OR “Health Education”[Mesh] OR “Patient Education as Topic”[Mesh] OR “Health Literacy”[Mesh] OR “Health Services for Persons with Disabilities”[Mesh] OR “Health Services for the Aged”[Mesh] OR “Health Services, Indigenous”[Mesh] OR “Culturally Competent Care”[Mesh] OR prevention[tiab] OR prevent[tiab] OR preventing[tiab] OR health service*[tiab] OR screening[tiab] OR health promotion[tiab] OR health education[tiab] OR patient education[tiab] OR health literacy[tiab] OR health care[tiab])
AND	(“Health Communication”[Mesh] OR “Reminder Systems”[Mesh] OR “Counseling”[Mesh] OR “Communications Media”[Mesh] OR “Motivation”[Mesh] OR “Information Dissemination”[Mesh] OR “Consumer Health Information”[Mesh] OR “Pamphlets”[Mesh] OR “Information Literacy”[Mesh] OR “Teaching Materials”[Mesh] OR intervention[tiab] OR interventions[tiab] OR health communication[tiab] OR communication media[tiab] OR reminder system*[tiab] OR counseling[tiab] OR counselling[tiab] OR health information[tiab] OR information dissemination[tiab] OR information literacy[tiab] OR teaching material*[tiab] OR pamphlet[tiab] OR pamphlets[tiab] OR booklet[tiab] OR booklets[tiab] OR leaflet[tiab] OR leaflets[tiab] OR flyer[tiab] OR flyers[tiab] OR poster[tiab] OR posters[tiab] OR brochure[tiab] OR brochures[tiab] OR access[tiab] OR communication strategy[tiab] OR communication strategies[tiab] OR strategy[tiab] OR strategies[tiab] OR audio*[tiab] OR video[tiab] OR videos[tiab] OR dvd*[tiab] OR compact disc*[tiab] OR cd[tiab] OR cds[tiab] OR “Multimedia”[Mesh] OR multimedia[tiab] OR multi-media[tiab] OR “Telecommunications”[Mesh] OR “Internet”[Mesh] OR internet[tiab] OR web[tiab] OR website*[tiab] OR online[tiab] OR electronic mail*[tiab] OR email*[tiab] OR mail*[tiab] OR “Blogging”[Mesh] OR blog*[tiab] OR weblog*[tiab] OR podcast*[tiab] OR portal*[tiab] OR computer program*[tiab] OR computer mediated[tiab] OR computer based[tiab] OR computer assisted[tiab] OR “Correspondence as Topic”[Mesh] OR telephon*[tiab] OR phone[tiab] OR phones[tiab] OR text messag*[tiab] OR sms[tiab] OR facilitator[tiab] OR facilitators[tiab] OR facilitate[tiab] OR motivation[tiab] OR motivators[tiab] OR motivational strategy[tiab] OR motivational strategies[tiab] OR enablers[tiab] OR promotional[tiab] OR beneficial[tiab] OR helpful[tiab] OR fostering[tiab] OR advantageous[tiab] OR barrier[tiab] OR barriers[tiab] OR barricade[tiab] OR impeding[tiab] OR hindering[tiab])
AND	(“2008/01/01”[EDAT] : “3000”[EDAT])

### Data management

The search results will be uploaded and managed using Microsoft Excel. A PRISMA flow diagram will be used to summarize and visualize study selection.

### Study selection

Two reviewers will independently screen titles and abstracts of search results against the inclusion criteria. In the next step, we will screen full-text reports for potentially eligibility. Full texts will be screened independently by two reviewers. Any disagreement will be resolved by discussion and consensus. The reasons for exclusion in full text will be documented. A list of excluded studies will be provided. The corresponding authors of eligible articles will be contacted for clarification where necessary. If the corresponding author cannot be reached, we will report this in the scoping review.

### Data extraction

A standardized extraction form will be developed for this review. The data extraction form will be piloted on a sample of five articles by the reviewers involved in the scoping review and will be assessed for completeness and applicability. Based on the pilot testing, any modifications to the standardized data extraction form needed will be undertaken to ensure the data necessary to address the research questions are obtained. The extraction form will contain general study characteristics and communication strategies. We will also extract the diabetes type, which will allow us to perform subgroup analyses in case the communication strategies differ between T2DM and GDM in any way. If possible, we will try to categorize the identified communication strategies. Data will be extracted by one reviewer and checked by another. Disagreements will be resolved through discussion and consensus.

### Data items

The preliminary data-extraction categories will be derived from our overarching research question. The following data will be collected:

- Study characteristics (e.g., country, setting, publication date, number of participants, target disease, study design/method)
- Patient’s characteristics (e.g., age, gender, affiliation to vulnerable group)
- Inclusion/exclusion criteria
- Communication strategies
- Implementation factors

### Risk of bias

As this is a scoping review, there will be no risk of bias assessment. This is consistent with guidance on the conduct of scoping reviews [9].

### Data synthesis

We will use Arksey and O’Malley’s methods [9] of reporting and provide a descriptive analysis of the extent, nature, and distribution of the studies included in the review as well as a narrative, thematic summary of the data collected. This will be achieved by summarizing the literature according to the types of vulnerable groups, communication strategies, comparators, implementation factors and outcomes identified. We aim to map the research landscape in this area. This will be facilitated by some form of visual representation of the data to map the extent, range, and nature of research in this area. Data will be charted, categorized, and summarized. We will report quantitative (e.g., frequency) and qualitative results. Furthermore, we will seek to explore similarities and differences, both within and between studies, to identify patterns and themes and to postulate explanations for findings. By doing so, we will also consider the robustness of the included studies themselves by reporting on the overall strength of and confidence in the findings. If possible, we will stratify our results by vulnerable groups.

## Discussion

The main aim of this review is to identify and describe communication strategies for the prevention of T2DM/GDM in vulnerable groups. The results will be used to inform the “National education and communication strategy on diabetes mellitus in Germany”. In particular, the results will be discussed in focus groups of experts to develop recommendations for communication strategies targeting vulnerable groups.

As this review is part of the “National awareness and prevention strategy on diabetes in Germany” conducted by the Federal Centre for Health Education and the Federal Ministry of Health, there is a narrow time frame for completing the report, and therefore, we have to limit the publication date. However, there might be too many differences in communication strategies due to digitalization. The results of this review will be used to make appropriate recommendations on the development of preventative measures targeting vulnerable groups which could be used in different German healthcare settings. Another strength of this study will be the systematic search for all published literature on that topic. As this review is part of the overall project commissioned by the Federal Centre for Health Education, it will have national coverage in improving health education.

## Abbreviations

GDM: Gestational diabetes
PPC: Population, Concept, Context
T2DM: Type 2 diabetes mellitus
WHO: World Health Organization
